# Application and Technique of Liquid Crystal-Based Biosensors

**DOI:** 10.3390/mi11020176

**Published:** 2020-02-08

**Authors:** Chonglin Luan, Haipei Luan, Dawei Luo

**Affiliations:** 1 School of Applied Chemistry and Biotechnology, Shenzhen Polytechnic, Shenzhen 518055, China; 2 School of Dentistry, University of Detroit Mercy, Detroit, MI 48208, USA

**Keywords:** liquid crystal biosensor, signal amplification technique, liquid crystal cell, virus, bacteria, protein, nucleic acid, small chemical molecules, metal ions

## Abstract

Liquid crystal biosensors are based on changes in the orientation of liquid crystal molecules induced by specific bonding events of biomolecules. These biosensors are expected to serve as a promising system to detect biomolecules, biomolecular activity, and even small chemical molecules because they are inexpensive, sensitive, simple, effective, and portable. Herein, we introduce the principle and fabrication of liquid crystal biosensors and review the research progress in signal-amplified technology for liquid crystal sensing and its application in the detection of viruses, bacteria, proteins, nucleic acids, and small chemical molecules. In addition, the current theoretical and practical issues related to liquid crystal biosensors were investigated.

## 1. Introduction

As a unique material, liquid crystals (LCs) flow readily like a liquid and maintain optical anisotropy similar to that of crystals. Within the range of the melting point and clearing point temperatures, LCs have multiple intermediate phases that are distinguished by their molecular orientations. The changes in temperature as well as changes in topography and chemical structure will affect the short-range interaction and long-range orientation sequences of LCs, resulting in orientation transitions. Therefore, LCs can be used as materials that respond to the presence of foreign species or the occurrence of binding events in their vicinity [1,2,3].

In 1998, the Abbott research group first used LCs as a signal converter to convert the antigen-antibody immunoreaction at the LC–solid interface into an LC optical response signal. This study wrote a new chapter for LC biosensors [4]. In 2002, the Abbott team conducted groundbreaking research, finding that sodium dodecyl sulfonate (an amphiphilic molecule containing long alkyl chains) adsorbed on the LC–aqueous interface could induce LCs to adopt a vertical, ordered arrangement. This discovery laid the foundation for the development of LC–aqueous interface biosensors [5].

As a result of in-depth research in recent years, LCs have been developed as a new type of signal converter for biological and chemical molecule sensing. This emerging LC-based sensing method provides a new visual detection technique and broadens the range of application of LCs.

## 2. Liquid Crystal (LC) Sensing Mode and Principle

**Figure 1:**
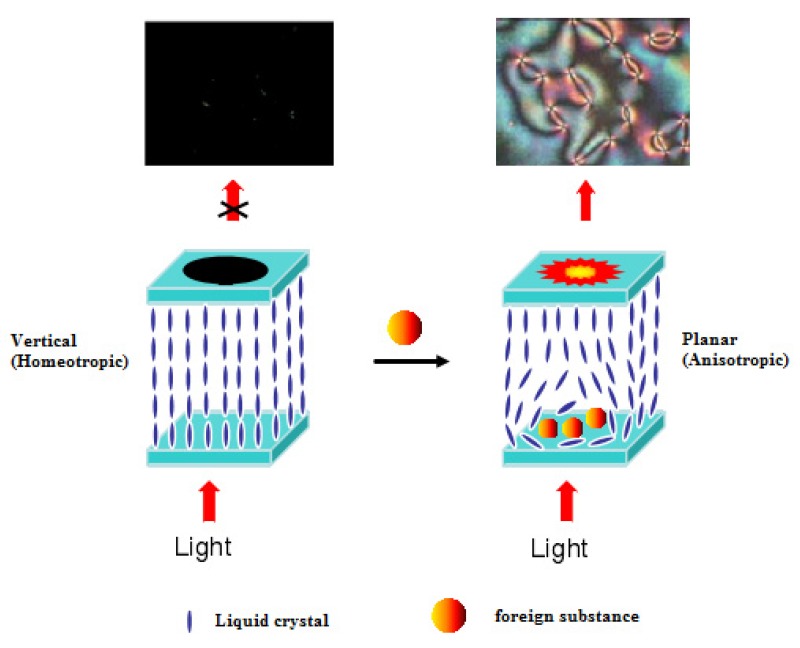
Schematic diagram of liquid crystal biosensor detection.

LC sensing is based on the birefringence (i.e., optical anisotropy) of LCs. The schematic diagram of liquid crystal biosensor detection is shown in Figure 1. If LCs are aligned in a vertical orientation and the direction of polarized light, which can pass through the liquid crystal layer but cannot pass through the polarizer, is parallel to the LCs, an all-black pattern will be observed under a polarized optical microscope. If the alignment of LCs is parallel or oblique, the birefringence of the incident polarized light on the surface of the LCs will be decomposed into two linearly polarized light beams with different propagation directions. This partially polarized light can penetrate the polarizer; thus, an image with a bright or rich color or a characteristic texture can be observed. Currently, LC sensing can be classified into two categories: LC–solid interface sensing and LC–aqueous interface sensing.

### 2.1. LC–Solid Interface Sensing and Its Principle

**Figure 2:**
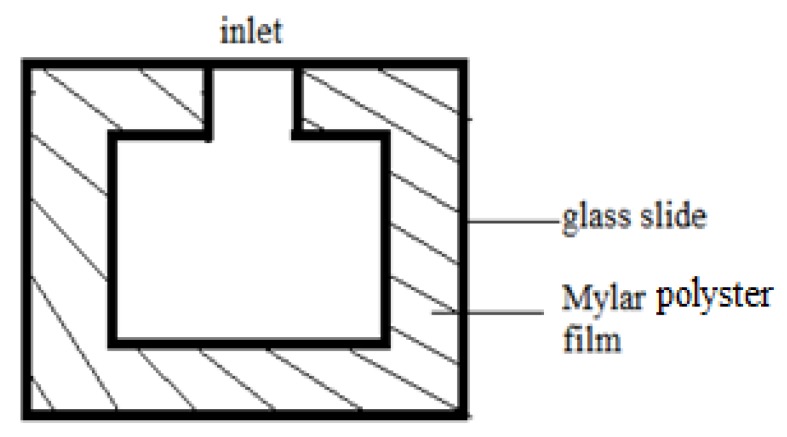
Cell for LC–solid interface sensing.

LC–solid interface sensing requires an LC cell. Usually, the cell is made up of cover and bottom microscopic glass slides together with a Mylar polyester film spacer, with a convex cavity cut in the middle of the film (see Figure 2) and an inlet on one side. The other three sides are usually fixed with binder clips.

**Figure 3:**
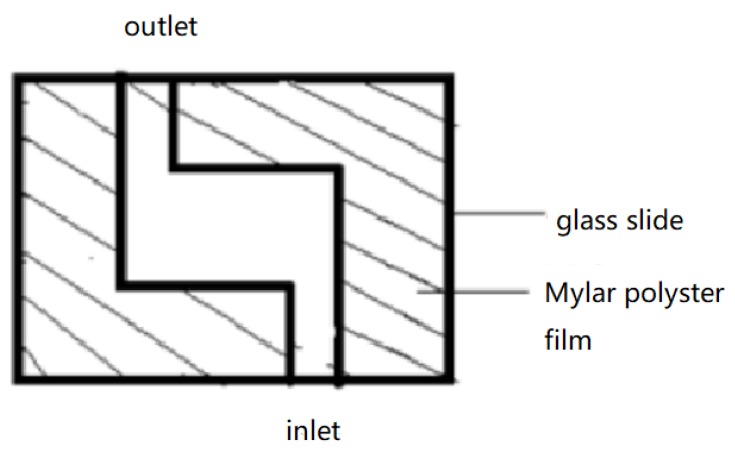
‘L’-shaped cell for LC–solid interface sensing.

The LCs are heated to 40–50 °C. At this temperature, the LCs transform from a solid state into a clear and transparent liquid state. The LCs are then injected by microsyringe into the inlet with a thermostat, filling the cavity quickly due to capillary action. After the LCs are added, the newly prepared liquid crystal sensor is naturally cooled to approximately 25 °C for observation. However, such an operation often results in air bubbles in the cavity. To address this issue, the Mylar film is cut into two centrosymmetric ‘L’ shapes to form an inlet and an outlet in the bottom and top edges, respectively (see Figure 3). This structure helps to expel air bubbles from the outlet on the top edge.

**Figure 4:**
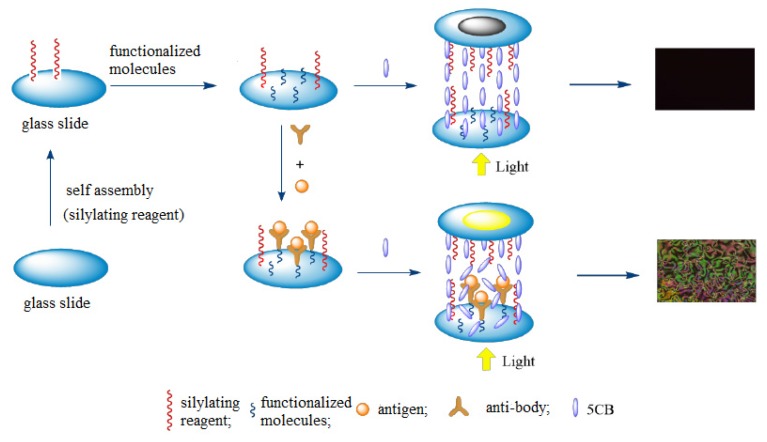
Diagram of the LC–solid interface sensing principle.

The principle of LC sensing is as follows (see Figure 4). First, an assembled membrane is formed to induce alignment of the LC molecules in an orderly and consistent orientation by modifying the cover and bottom glass microscope slides. Then, the assembled membrane on the surface of the bottom glass slide is coated with the substrates or various types of functionalized molecules. Next, the target molecules are selectively and reversibly bonded to the surface of the assembled membrane by utilizing the strong interaction (such as acid–base interaction, coordination interaction, and antigen-antibody binding) between the target molecules and the substrates or functionalized molecules. The nontarget molecules do not have such strong competitive interactions.

Since the interaction between LCs and the assembled membrane is disrupted, the consistent orientation of the LCs is disturbed, which induces a transition of the color and brightness of the LCs viewed under a polarized optical microscope. The change in color indicates the presence of target molecules, and the change in brightness indicates the concentration of the target molecules.

### 2.2. LC–Aqueous Interface Sensing and Its Principle

The LC–aqueous interface sensor is mostly composed of a piece of coated glass microscope slide, TEM mesh (copper or gold mesh), LC materials, and buffer solution for analyzing the samples.

**Figure 5:**
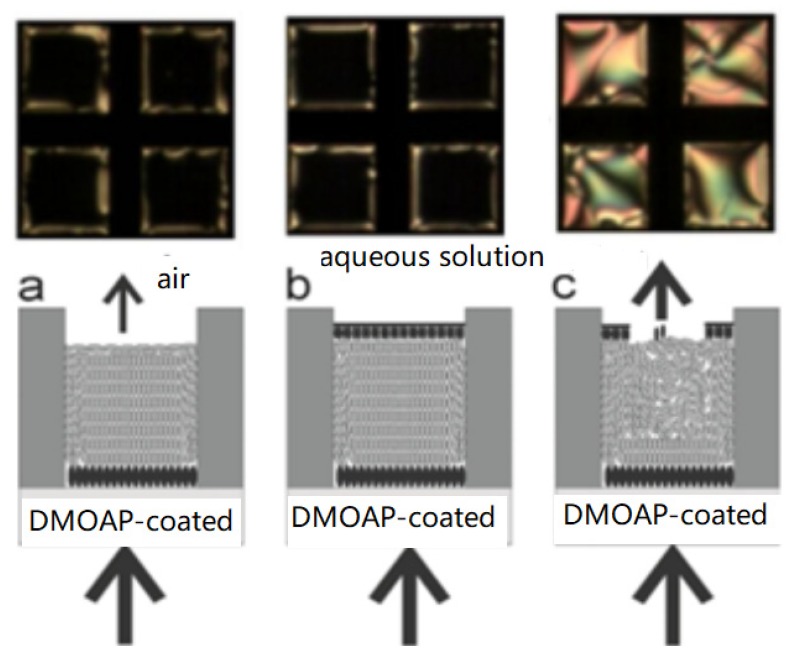
Schematic representation of an LC-based sensor consisting of a gold grid compartment, a silanized glass substrate and LCs; the arrows indicate the transmission of light: (**a**) LCs appear aligned near the substrate and slightly disordered near the air interface. (**b**) LCs exhibit homeotropic alignment near an aqueous environment, mediated by a lipid layer. (**c**) Destruction of the lipid layer results in disorder of the LCs [6].

The TEM mesh is first placed on the surface of a glass slide that has been coated with an alkylating agent (such as *N*,*N*-dimethyl-*N*-octadecyl-3-aminopropyltrimethoxysilyl chloride, DMOAP), as shown in Figure 5. Then, LCs are dropped into the mesh by capillary action, forming a liquid crystal layer approximately 20 μm thick, on which surfactants such as phospholipids are added to form a self-assembly monolayer at the LC–aqueous interface to induce the LCs to align vertically (homeotropic), which leads to the LCs showing an all-black image. Once the target molecules (phospholipase) are added, phospholipase can catalyze the hydrolysis of phospholipid molecules and destroy the self-assembly of these molecules at the LC interface. The arrangement of LCs is changed (to be planar), and the optical image of LCs changes from black to bright, thus realizing the detection of phospholipase activity [6].

The Abbott group did lots of pioneer work at the LC–aqueous interface. They studied the effects of amphiphilic molecules [6,7], polymer electrolytes [8,9], ionic surfactants [10,11] biomolecules [12] and other substances on the orientation of liquid crystal arrangements at the liquid crystal aqueous interface. It is primary work for the research of LC–aqueous interface sensing.

## 3. Modification for LC substrates

Generally, the LC biosensor first needs to arrange the LC molecules in order. Since the orientation of LCs near the surface of the substrate is easily affected by the chemical composition, topographical structure, and energy of the surface, changes in the chemical composition and morphology will disrupt the orientation balance of LCs, affecting their optical imaging. Therefore, modification of the substrate for LC biosensors is very important. Currently, the methods of constructing LC sensing substrates mainly include constructing a gold film, a mechanically rubbed film, and a silylating reagent self-assembly film.

The gold film method [13,14] involves the use of a vacuum electron beam coating machine that plates a gold film with nanogrooves on the slide base at a certain angle (typically 45° ± 0.5°). Then, a single-molecule self-assembled membrane containing an alkyl chain (C atomic number less than 10) on the surface of the gold film is formed through Au-S bonding. The synergistic effect of the gold film and self-assembled membrane is used to induce parallel or vertical alignment of the LCs. Since this method requires a very high skill level, to a certain extent, its application is limited.

The rubbed film method [15] involves a traditional LC alignment, which is achieved by the use of materials (such as nylon, cotton wool, or fiber) to rub against the membrane so that directional scratches or grooves, which have a uniform anchoring effect, are generated. This method is simple but also has some disadvantages, such as the easy generation of dust particles, detachment of foreign matter during the friction process, and difficulty in controlling the uniformity of the friction.

The silanization reagent assembly method can induce the vertical or parallel alignment of LCs by controlling the length of the alkyl chain, which is a simple, effective, and widely used technique. Dimethyl octadecyl (3-[trimethoxysilane] propyl) ammonium chloride (DMOAP), for example, has a long alkyl chain. After being adsorbed onto the substrate surface, DMOAP can induce LCs to arrange in an orderly manner along the alkyl chain, resulting in an all-black background, so it is often used for substrate modification. In general, functional molecules such as 3-aminopropyl triethoxysilane (APTES) need to be introduced onto the substrate surface to immobilize the target molecule.

Price et al. [16] found that the color and brightness of the LC film increases linearly with increasing wettability of the substrate surface. The smaller the contact angle is, the more easily the LC molecules approaches a parallel orientation, and the critical transition value is ~64°.

Nakagawa et al. [17] discovered that if the length of the molecular chain on the surface of the self-assembly membrane is greater than or equal to a certain critical value, the self-assembly membrane is formed in an orderly arrangement due to the action of van der Waals forces between carbon chains. If the length of the carbon chain is shorter than the critical value, the van der Waals force generated is small, and the molecules are easily tilted in the aggregation process, so forming an ordered self-assembly film is difficult. Therefore, the silanization reagent containing a long alkyl chain can induce the vertical arrangement of LCs, while that containing a short alkyl chain cannot induce the vertical arrangement of LCs. According to the different properties of the silanization reagent, the critical values are different.

Porte’s study [18] demonstrated that the length of the alkyl chain (n) affects the alignment orientation of n-(4-methoxyphenylene)-4-butylaniline (MBBA) molecules, a kind of nematic LCs. When 6 ≤ n ≤ 10, MBBA molecules were arranged in an oblique manner; when 12 ≤ n ≤16, LC molecules were arranged vertically.

According to the different properties of silanization reagents, the Abbott group selected OTS to construct different sensitive substrate membranes [1,5,19]. Yang’s team used DMOAP as the sensitive substrate membrane to construct an LC sensor [20]. To facilitate the assembly of biological molecules on the surface of the substrate, these researchers also used the mixed assembly mode of DMOAP/TEA [21] or DMOAP/APS [22] to construct a membrane that could not only induce vertical arrangement of the LCs, but also facilitate immobilization of biological molecules.

In the year of 2002, the Whitesides group [23] produced metallic half-shells (like a bowl) with nanometer-scale dimensions. The unique structure of these half-shells provides a good potential uses in the field of LC sensing. In 2011, a new method for modification of LC substrate was developed by the Zhao group. They synthesized hydrophobic NiNPs (NiNBs) with a bowl structure by magnetic self-assembly with polyvinylpyrrolidone (PVP) as a stabilizer and then synthesized Ni nanospheres (NiNS) [24], finally doping them with LC molecules. The effect of the NiNP shape on the orientation of the LCs was studied [25]. It was found that NiNPs with a bowl structure can induce the planar orientation of LC molecules, while NiNS can induce the vertical orientation of LC molecules.

## 4. Signal Amplification for LC Sensing

LC biosensors have become a new research focus in recent years because of their advantages of low consumption, fast and simple operation, and visualization of optical signals. However, the sensitivity of the sensor is not always enough, so it is necessary to improve the sensitivity of LC biosensors with the help of signal amplification technology.

### 4.1. Signal Amplification Techniques Based on Nanomaterials

Gold nanoparticles have the properties of a large specific surface area, high surface free energy and good biocompatibility. Thus, they can significantly increase the number of biomolecules immobilized and the size of the complex to carry out signal amplification. Additionally, they have the advantages of a simple synthesis process, good chemical stability and firm binding with biomolecules containing sulfhydryl or amino groups.

**Figure 6:**
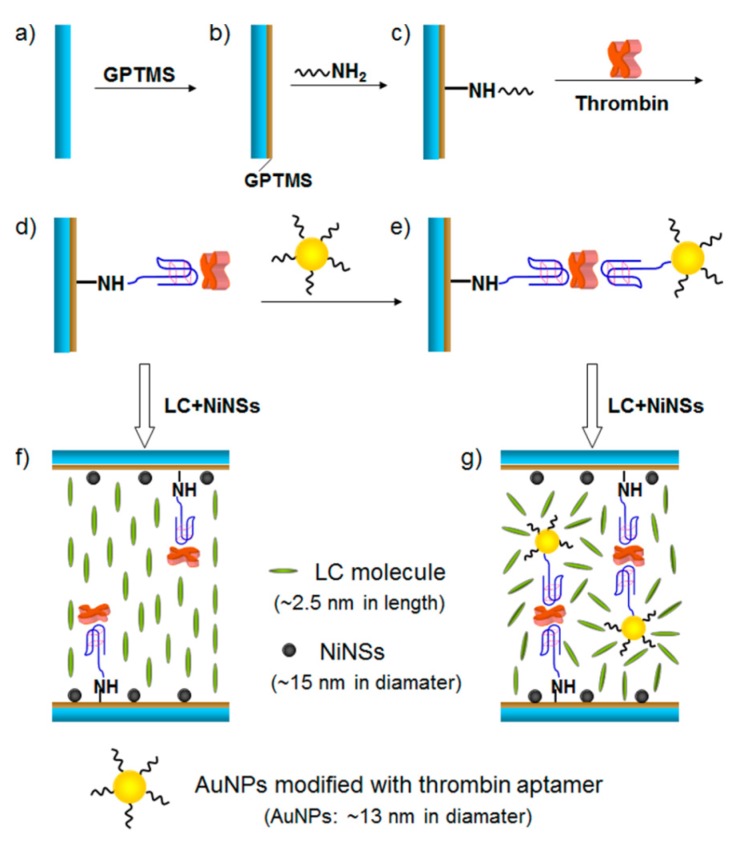
Schematic illustration of the detection method and preparation steps for the NiNS-based thrombin LC sensor: (**a**) cleaned glass slide; (**b**) self-assembled GPTMS film; (**c**) immobilization of the antithrombin aptamer; (**d**) thrombin addition and binding with the aptamer; (**e**) binding with Apt-AuNPs; (**f**) orientation of the LC mixture (5CB doped with 0.01 wt % NiNS) in the LC cells assembled with thrombin; (**g**) orientation of the LC mixture in the LC cells assembled with Apt-AuNPs through a sandwich format [26].

Using gold nanoparticles as a signal amplifier, Zhao [26] proposed a new LC-based sensing approach for the detection of thrombin. The nickel nanospheres (NiNSs) were used to induce LCs to be vertical alignment. The specific interaction between thrombin and its binding aptamers was employed to develop the thrombin LC sensor. Through the two binding sites of thrombin, which links the monodispersed aptamer and aptamer-functionalized AuNPs (Apt-AuNPs) together to make the aggregation. The procedure is shown in Figure 6.

In this study, a single AuNP was loaded with ~80 aptamer units per particle [27]. The disruption to the LC orientation is enhanced by the gold nanoparticles (AuNPs)−aptamer conjugation, which increased the disruption to the LC orientation by utilizing gold nanoparticles’ robust nature, stability, and high ratio of surface area to volume that causes amount of adsorption of different directional thrombin aptamer, and turned to amplification of the optical signals under crossed polarizers. The detection of limit for thrombin is as low as 0.06n mol·L^−1^.

**Figure 7:**
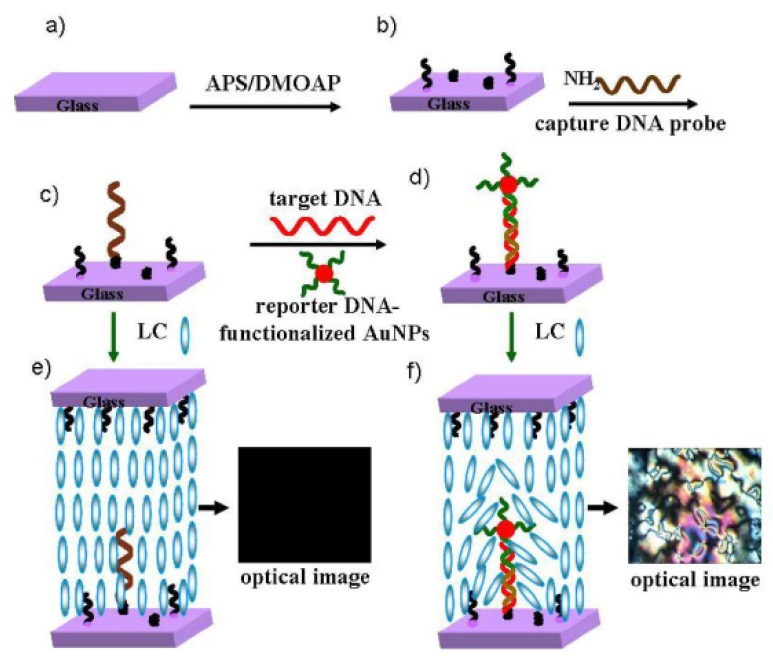
Principle of of LC biosensor based on gold nanoparticles signal enhancement. (**a**) cleaned glass slide; (**b**) assembled APS/DMOAP substrate; (**c**) fixed capture DNA probe substrate; (**d**) occurrence of binding events; (**e**) optical image of LC sensing cell before binding events; (**f**) optical image of LC sensing cell after binding events, disruption of the homeotropic LCs [28].

Wu’s research group [28] also amplified the signal by introducing AuNPs. A highly sensitive liquid crystal biosensor based on gold nanoparticles signal enhancement is fabricated. As shown in the Figure 7, the substrate surface was first assembled with dimethyl octadecyl aminopropyl trimethoxymethylsilylammonium chloride (DMOAP) and aminopropyl trimethoxysilane (APS) silanization reagents to provide a sensitive film without interference background and convenient for immobilization of biomolecules, and then the capture probe (T1, TGGAAAATCTCTAGCAGTCGT-(CH_2_)6-NH2) was fixed on its surface. When the target molecule (T2, ACTGCTAGAGATTTTCCACACTGAAAAGGGTCTGAGGGA) existed, its one end could be crossed with the capture probe T1, and the other end could be crossed with the gold nanoparticles labeled signal probe (T3, SH- (CH_2_) 6-ATGTCCCTCAGACCCTTT). Because a large number of signal probes can be fixed on the surface of AuNPs, the introduction of AuNPs can significantly change the topography of the substrate surface and enhance the effect on the orientation of LCs, the detection limit of 0.1p mol·L^−1^ can be obtained.. The method is simple, sensitive, and accurate.

**Figure 8:**
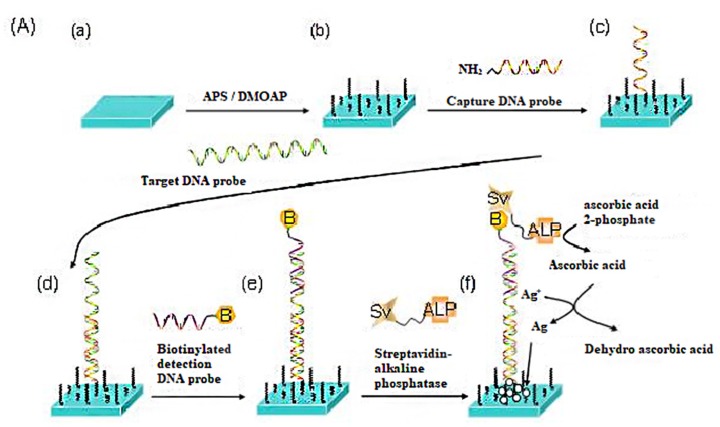
Stepwise assembly of the signal-enhanced LC DNA biosensing substrate based on enzymatic silver deposition: (**a**) cleaned glass slide; (**b**) self-assembled APS/DMOAP film; (**c**) immobilization of cap-ture DNA Probe; (**d**) hybridization with target DNA; (**e**) hybridization with biotinylated detection DNA probe; (**f**) association with streptavidin alkaline phosphatase and reduction of silver ions by ascorbic acid [29].

Furthermore, they utilized a biotin-streptavidin system and combined enzyme catalysis with Ag deposition for signal amplification. The process is depicted in Figure 8. Firstly, a chemically functionalized surface on a plane glass slide is obtained by self-assembling a APS/DMOAP film. Then, the DNA immobilization is realized by binding a capture DNA probe to the APS/DMOAP film through a cross-linker, followed by hybridizations of a target DNA and a biotinylated detection DNA probe. Subsequently, the streptavidin-alkaline phosphatase (Sv-ALP) is bound to the biotin of the detection probe and then catalyzes the hydrolysis of ascorbic acid 2-phosphate (AA-p) to form ascorbic acid. The latter, in turn, reduces the silver ions in solution to form the deposition of metallic silver on the substrate surface. The results showed that this method is highly sensitive for the detection of DNA compared with the method of bonding biomolecules directly because enzyme-catalyzed Ag nanoparticles with larger geometric dimensions can greatly change the topological structure of the surface of the self-assembled monolayers to enhance the optical signal [29].

**Figure 9:**
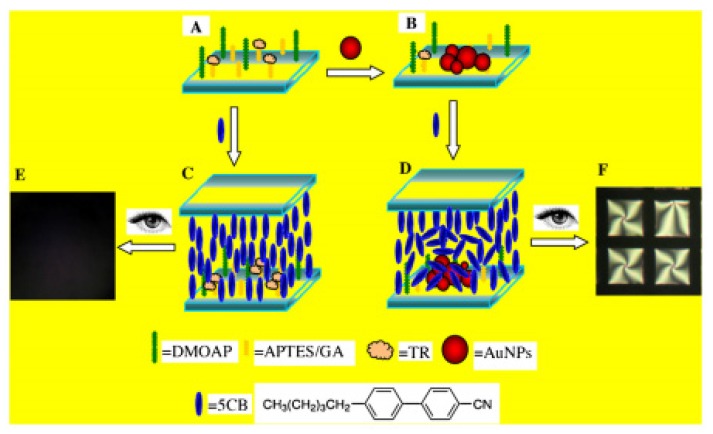
Illustration diagram of detection strategy of LC biosensor: (**A**) cimmobilization of TR; (**B**) deposition of AuNPs through the enzymatic process; (**C**) homeotropic orientation of 5CB in the cells without AuNPs on glass slides; (**D**) the disrupted orientation of 5CB in the cells with AuNPs deposited on glass slides. Photograph under polarized light microscope (**E**,**F**) [30].

Xiong [30] reported an LC biosensor based on enzyme-mediated AuNP growth without adding AuNPs as crystal seeds for the detection of tyrosine (Tyr), see Figure 9. Tyrosinase (TR) can catalyze the hydroxylation of Tyr to levodopa (L-Dopa). AuCl_4_ in L-Dopa solution was reduced to AuNPs depositing on the glass surface, as time went by aggregates of nanoparticles were formed. The amount of these particles of various shapes and sizes could greatly change the surface topology and induce a homeotropic-to-tiled transition of the LC molecules, resulting in a significant change in corresponding of optical appearances under the crossed polarized light. The detection concentration of Tyr can be as low as 6 × 10^−7^ mol·L^−1^. Compared with the direct introduction of AuNPs, the enzyme-mediated AuNP growth method does not need the presynthesis of AuNPs.

The cases utilizing nanomaterials for signal amplification considered above focused on a LC–solid interface sensing system, but some of them may be useful in LC–aqueous interface sensing.

### 4.2. Signal Amplification Technique Based on DNA Hybridization

**Figure 10:**
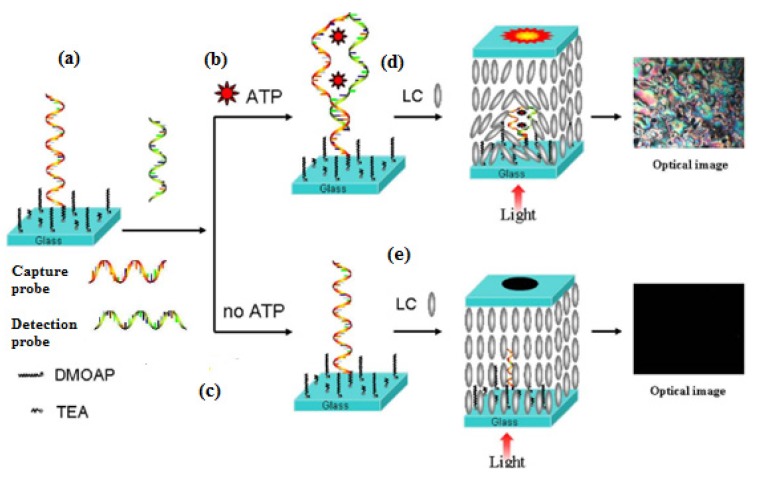
Stepwise assembly of the LC biosensing substrate based on ATP aptamer. (1) immobilization of capture DNA probe on the TEA/ DMOAP mixed SAM modified glass slide (**a**); (2) hybridization with detection DNA in the presence of ATP (**b**) or in the absence of ATP (**c**); (3) The corresponding 5CB LC cells (**d**,**e**) fabricated with the DMOAP-coated glass slides (upper) and the modified slides (**b**,**c**; lower) [31].

The double-helix structure of double-stranded DNA has a certain steric hindrance, which can effectively disrupt the orientation of the LCs, and therefore, the small molecules can be detected by using a nucleic acid chain hybridization reaction as a means of signal amplification. The Wu research group [31] developed an ‘aptamer-target molecule-aptamer’ ‘sandwich’ biological sensor based on LC sensing to detect adenosine triphosphate (ATP). As shown in Figure 10. The single-stranded ATP aptamer is split into two fragments in this method, one of which, as a capture probe, is covalently immobilized on the surface of a slide substrate modified by a TEA/DMOAP assembly film, while the other is used as the detection probe. When ATP is present, two sections of the split nucleic acid aptamer combine into a double-stranded structure by folding with ATP. This binding event leads to a considerable enhancement in the optical signal of the LC biosensor—due to the space size—from small to large, which can effectively disrupt the orientational arrangement of LCs, resulting in the corresponding changes in optical images under the crossed polarized light. The detection limit of ATP is 10 nmol·L^−1^.

**Figure 11:**
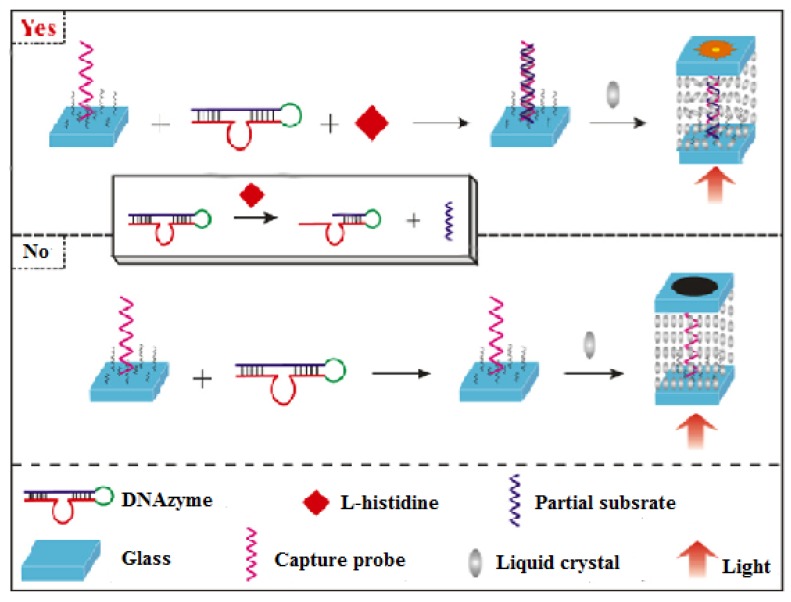
Description of the high-sensitivity DNAzyme-based LC biosensor for L-histidine. Only L-histidine can cleave the DNAzyme and release partial substrate, which hybridizes with the capture probe forming ds-DNA on the glass slide. The ds-DNA induces a homeotropic-to-tiled transition of the LCs, resulting in a birefringent texture of the optical image for LC cell [32].

They also utilized L-histidine- to cleave DNAzyme to expose some of its fragments, causing its hybridization with the DNA capture probe to form double-stranded DNA. The procedure is depicted in Figure 11. An optimal amount of capture probe is firstly bound to the glass slide, which changes the surface topology as little as possible and shows a zero-background for the sensing system. Then, the DNAzyme molecule is cleaved by the target, L-histidine, a partial substrate strand is produced, which in turn can hybridize with the capture probe, forming a DNA duplex. Due to the duplex being able to seriously disrupt the LC molecular orientation, this biosensor realized the detection of L-histidine with a detection of 50 nmol·L^−1^ [32].

**Figure 12:**
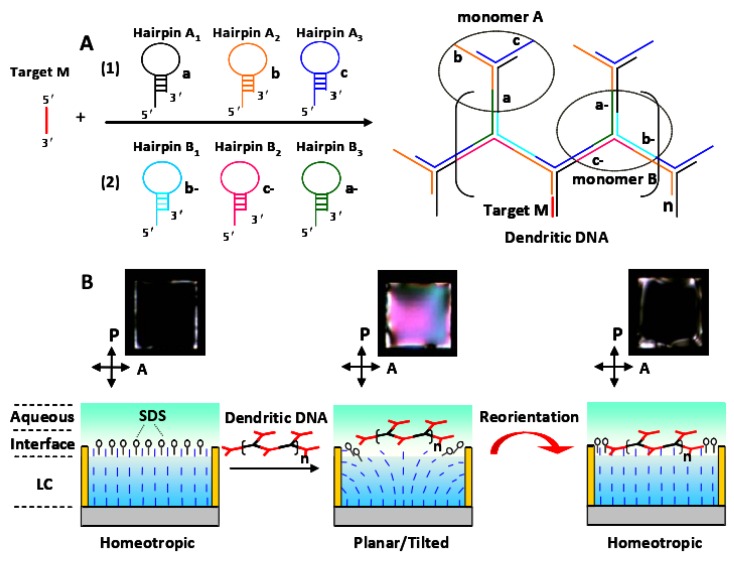
Schematic of liquid crystal biosensing method based on DNA dendrimer signal enhancement. (**A**) Target M initiates a hybridization chain reaction of hairpin probes to form dendritic DNA polymers; (**B**) the process of changing the orientation of liquid crystals when dendritic DNA polymers are adsorbed at the liquid crystal–aqueous interface [33].

Tan [33] proposed a new signal-enhanced LC biosensor based on target-triggering DNA dendrimers for the highly sensitive detection of p53 mutation gene fragments. The schematic of liquid crystal biosensing method based on DNA dendrimer signal enhancement is shown in Figure 12 In this study, the mutant-type p53 gene segment was the target to trigger the formation of DNA dendrimers from hairpin DNA probes by a hybridization chain reaction. The shift of homologous to tilted alignment of liquid crystal 5CB is related to the electric dipole coupling of adsorbed DNA with the LCs, while the reorientation of the liquid crystal 5CB is not only restricted by DNA conformation but also induced by the internal electric field after hybridization. Specifically, the unique optical reorientation signal induced by the DNA dendrimer to texture or a dark frame can be used as a characteristic signal to differentiate between target concentrations from 0.008 nmol·L^−1^ to 8 nmol·L^−1^. This finding shows that DNA dendritic molecules can be used as a good signal enhancer to induce reorientation of the LC water interface, which provides a sensitive method for the detection of specific DNA sequences without marking. This LC biosensor strategy is expected to be a promising means to study the interaction of various DNA (including aptamers), such as base-pair interactions, metal-based interactions, and protein aptamer binding events.

## 5. Application of LC Sensing

In the past two decades, the application of LC sensing has made much progress in cancer cell diagnosis and the detection of viruses, bacteria, proteins, DNA, glucose, metal ions, and other small molecules.

### 5.1. Cancer Cell Diagnosis and Detection of Virus and Bacteria

**Figure 13:**
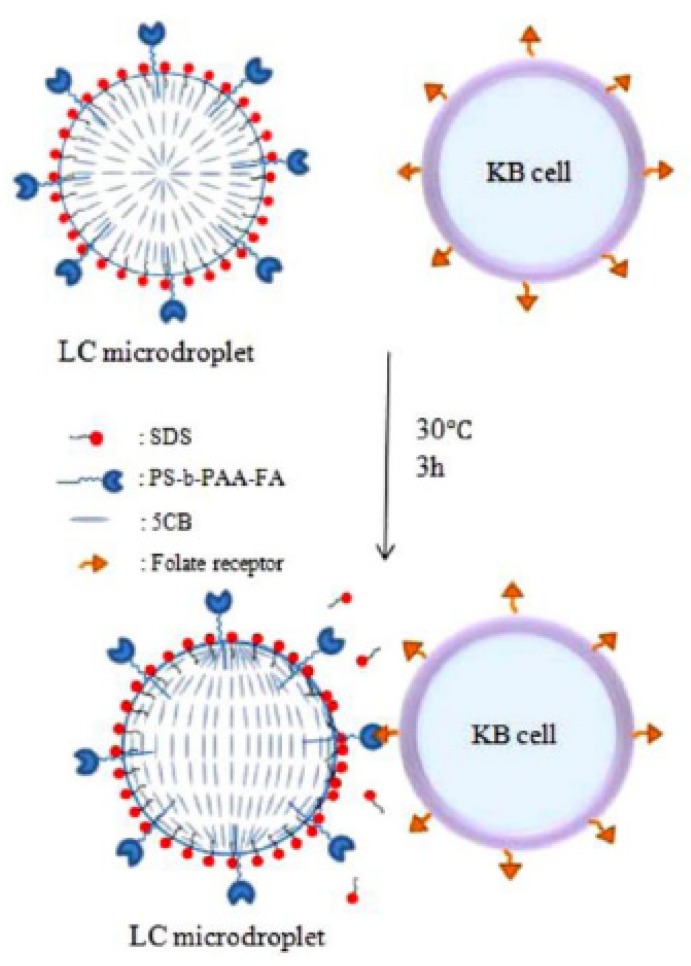
KB cancer cell (mouth epidermal carcinoma cell) interactions with LC micro droplets containing folic acid-conjugated PS-b-PAA [34].

The Park group [34] has utilized configurational transitions of a 5CB microdroplet emulsion (LCEM) from R (radial) to B (bipolar) to diagnose KB cancer cells because KB cancer cells interact with the LCEM. This facial phenomenon was produced by using folic acid-conjugated polystyrene and sodium dodecyl sulfate as a mediator to induce high selectivity and sensitivity. KB cancer cell selectivity has been considered because of the presence of a cancer cell folate receptor-specific ligand from KB. The diagram of the process is shown in Figure 13.

Similarly, Choi [35] developed LC microdroplets prepared for the detection of HepG2 cells in the presence of SDS as a mediator and β-galactose-conjugated PS-b-PAA (PS-b-PA-G) as a modifier of LC water interfaces. The orientational transition of LC microdroplets from R to B is due to the interaction of HepG2 cells with maltotriose-conjugated block copolymers. The detection limitation is 1.0 ± 0.1 HepG2 cells per μm^2^, with significantly high reproducibility (*p* < 0.05, *n* = 3).

Similarly, Sivakumar [36] also exploited LC emulsion droplets to detect and differentiate between Gram-positive and Gram-negative bacteria as well as between enveloped and nonenveloped viruses. It was demonstrated that Gram-negtive bacteria and enveloped viruses can cause a configuration transition of LC molecules from B to R. This transition was considered to be consistent with the lipid transfer from the organisms to the LC droplet interfaces. This sensing scheme can be applied for rapid and sensitive assays to screen a large number and variety of bacteria and viruses based on their structural features. Small numbers of *E. coli* (1–5) and low concentrations (10^4^ pfu·mL^−1^) of virus were actually detected by this method.

Xu [37] reported the binding events occurring at the PEI (poly(ethylene imine))-coated interfaces of the LC. The *E. coli* strain TOP10 induces a homeotropic orientation of LC by electrostatic attraction with PEI.

Similarly, Zafiu [38] reported a method for the detection of bacteria by utilizing the interaction of lipopolysaccharides (PPS) with liquid crystals, visualized in an LC-based sensing system. This LPS/LC combination as a sensing layer could interact with three different bacterial species, and the presence of bacteria was detected regardless of their viability with high sensitivity (minimum of 500 cells mL^−1^) and high efficiency (maximum detection time, 15 min). The read-out mechanism was proven to be adsorption of bacteria entities on the surface bound LPS molecules, with the LCs acting as an optical amplifier.

### 5.2. Detection of Protein and DNA

Enzymes, as a kind of protein, play an important role in life science. There are some studies focusing on their detection by LC-based sensing platforms. Hartono et al. [39] reported a new LC platform to analyze phospholipases based on molecular interactions between LCs and phospholipases at aqueous-LC interfaces. Three phospholipases—phospholipase A2, phospholipase C, and phospholipase D—were detected and further confirmed by incorporating phospholipase inhibitors in the LC-based sensing scheme. Such a sensing platform could provide a facile, label-free assay to characterize the presence and activities of phospholipase and provide the possibility to screen enzyme inhibitors.

Furthermore, Hartono and coworkers [3] adopted the same sensing scheme to complete the detection of beta-bungarotoxin, a phospholipase-like toxin, which is a protein toxin that shows phospholipase-like enzymatic activity, enabling the hydrolysis of organized and self-assembled structures such as cell membranes. This toxin hydrolyzes the phospholipase monolayer, which results in the orientational responses of LCs with a very low detection limit of less than 5 pg of the toxin. This sensing platform was proven to be a simple and cost-effective method that could be extended to screen many compounds to find inhibitors against such toxins.

In another investigation, Hu and Jang [40] reported that lipase can catalyze the hydrolysis of triacylglycerols to their various fragments. In these experiments, a self-assembled monolayer of surfactants at the LC–aqueous interface was formed by oleic acid, which was produced by the enzymatic reaction between lipase and glyceryl trioleate. Glyceryl trioleate-doped 5CB was used to indicate the transition of LCs from planar to homeotropic.

Urease [41], trypsin [42], catalase [43], and acetylcholinesterase [44] are known to be very important enzymes in clinical testing. Researchers have reported a few detection methods for these enzymes by means of an LC sensing scheme, which results in the transition of the configuration of 5CB. The developed sensing platforms show great promise for label-free detection of them. However, many works are still waiting to bridge the gap between the fundamental detection principle and actual application.

Other proteins have also been studied by some researchers. Park et al. [45] have exploited the 5CB LC biosensor for the detection of bovine serum albumin (BSA), hemoglobin (Hb), and chymotrypsinogen (ChTg). The sensing principle was based on electrostatic interactions between proteins and polyelectrolytes, such as poly(2-dimethylamino)ethyl methacrylate-b-(4-cyanobiphenyl-4’-oxyundecylacrylate) (PDMAEMA-b-LCP). A change from H to P in the orientation of 5CB was observed at concentrations greater than 0.02 wt % within the pH range between the isoelectric point (pI) of BSA and the pKa of PDMAEMA. Hb and ChTg were also tested at different pH values by the same scheme mentioned above. A similar study was completed by using QP4VP-b-LCP, PSSNa-b-LCP, and PDADMAC as functional groups for the detection of proteins [46].

To detect DNA targets, Lai [47] reported a method involving the use of cholesterol-DNA probes at the LC–aqueous interface in the form of a self-assembled thin layer. The optical change in LC molecules was caused by a single-stranded DNA hybridization reaction.

The Park group [48] exploited the LC-based DNA biosensor. In this experiment, an LC-filled TEM grid cell was coated with the cationic surfactant dodecyltrimethylammonium bromide (DTAB). The DTAB-coated E7 (LC mixture) in the TEM grid (TEMDTAB) showed a homeotropic orientation, which converted to a planar orientation upon adsorption of the ssDNA probe. TEM/DTAB/DNA was then exposed to target ssDNA, which caused a configurational change in E7 from P to H that could be observed under a polarized optical microscope. A concentration of 2 μM ssDNA probe was chosen as the optimum concentration to react with more than 0.05 nM target ssDNA. This TEM/DTAB/DNA biosensor could distinguish target ssDNA from mismatched ssDNA and double-stranded DNA and was also applied to detect the genomic DNA of the bacterium *Erwinia carotovora* and the fungus *Rhizoctonia solani*.

In another study, the Abbott group [49] reported a general strategy to prepare chemically modified LCs to design biotic-abiotic interfaces. In this experiment, EG4 (oligomeric ethylene glycol) was functionalized with mesogens as a biological target. The sensing scheme can be applied to study various biological binding events at the interfaces, providing an exploration method for various biological functions.

### 5.3. Detection of Small Molecules

Glucose is the most widely distributed and important monosaccharide sugar in nature, occupying an important position in biology, and is the energy source and metabolic intermediate of living cells. Therefore, the detection of glucose is of great significance in life science. LC biosensor detection of glucose is usually based on the specific reaction between glucose oxidase and glucose.

The Park research group [50,51] exploited a glucose LC biosensor based on the principle mentioned above. A vertical-to-parallel alignment transformation of the LC 5CB was observed under a polarizing microscope. The results showed that the optimum fixed quantity of GOx was 780 μmol·L^−1^, the detection limit of glucose was 0.5 mmol·L^−1^, and the response time was less than 180 s. In subsequent research, this group fixed GOx and HRP on the PAA-b/LCP copolymer to establish a double enzyme-based glucose LC biosensor. The optimum molar ratio of GOx to HRP was 3:1. Compared with the only GOx-based biosensor, the glucose detection limit was reduced to 0.02 mmol·L^−1^ [52].

Bisphenol A (BPA) shows properties similar to those of hormones, which raises concerns about its content in consumer products and food containers. The Jang group [53] developed an LC biosensor for the detection of bisphenol A (BPA) based on the specific binding between BPA and its aptamer. In this experiment, the surface of the sensor was assembled with (3-aminopropyl)triethoxysilane and dimethyloctadec [3-(trimethoxysilyl)propyl]ammonium chloride, and the amino-aptamer was immobilized on this film via glutaraldehyde links. Once bisphenol A was added, a large aptamer-BPA complex was formed through a specific reaction, which disrupted the orientation of 4-cyano-4’-pentylbiphenyl in the film on this surface from homeotropic to random, causing an image change from dark to bright under a polarized optical microscope. The limit of detection of BPA is 600 pmol·L^−1^.

Niu et al. [54] proposed a biosensor for the cholic acid (CA) test based on the change in the configuration of the LC droplet. SDS was coated on the surface of 5CB liquid crystal droplets, which caused the LC droplets in PBS solution to adopt a radial configuration. As CA is easier to adsorb to the surface of the LC molecules, after the addition of CA, the CA molecule will compete with SDS for adsorption. In this case, the optical configuration of the crystal droplets is from a radial to a bipolar type structure. Detection of CA is achieved at 5 μmol·L^−1^ Compared with the prior LC film biosensor, the detection limit of this method is reduced by 2.4 times.

The detection of metal ions has always been an important topic in the field of analysis. A few researchers have proven that LC-based sensors are effective, rapid, inexpensive, and portable devices for metal ion tests.

Yang et al. [55] designed a novel LC biosensor for high-selectivity and high-sensitivity detection of heavy metal ions. The effect of DNA chain on the orientation of LC is not only related to the molecular weight and size of the chain, but also affected by the DNA chain spatial configuration. To improve the ability of the metal ion to interfere with the LC orientation, the sensor makes use of the DNA conformational change induced by the target to enhance the interference with the orientation of the LCs, thus amplifying the optical signal. Hg^2+^ is a typical heavy metal ion that has unique properties and can be specifically combined into two DNA thymosin (T) bases. In the presence of Hg^2+^, the specific oligodeoxyacid probe undergoes conformational changes from the hairpin structure to the duplex like complex, which cause the orientation of the LC molecules changes, thereby affecting the image of the LCs under a polarized light microscope. The results show that the sensitivity of the sensor is good, and the detection limit of Hg^2+^ is as low as 0.1 nmol L^−1^.

Recently, Singh et al. [56] developed an LC-based platform for the real-time detection of Hg^2+^ in water. In this system, amphiphilic potassium N-methyl-N-dodecyldithiocarbamate (MeDTC) was doped with 5CB and acted as a chelating ligand for Hg^2+^, which can induce the transition of LCs from dark to bright under polarized light. The detection limit was 0.5 μmol·L^−1^ for Hg^2+^.

More recently, Park et al. [57] proposed an LC sensor for the detection of Ca^2+^ in human saliva. In this experiment, PAA-b-LCP was functionalized on the interface of aqeous-5CB, wherein the LCP block was anchored to 5CB and the PAA block was complexed with metal ions in water. NaCl-treated cells exhibited homeotropic-to-planar orientational changes because Ca^2+^ replaced Na^+^ in the PAA chains, demonstrating successful noninvasive detection.

### 5.4. Other Sensing Techniques Based on LC

Conventional liquid crystal (LC)-based biosensing techniques are predominantly to utilize the property of optical anisotropy, or birefringence, in LCs to provide detection signals at a LC–solid or LC–aqueous interface. However, some innovative applications of the capacitance, electrooptical, and dielectric measurements of LCs have emerged. The Lee group [58] reported a LC-based capacitive biosensor, whose LC cell can be considered a parallel-plate capacitor. LCs were homeotropically or vertically aligned by DMOAP firstly. In the presence of BSA, a sufficient amount of immobilized BSA destroyed the anchoring strength of DMOAP, thus disrupted the homeotropic alignment of LCs. Therefore, an electric field is required to reorient LCs to the homeotropic state. It is assumed that the amount of BSA increased with the voltage required to redirect LC molecules to homeotropic alignment. According to this basic principle, the amount of BSA can be detected. Also, they exploited LC-based electro-optical biosensor [59] and LC-based dielectric biosensor [60].

These studies provide new approaches for the investigation and application of LC-based biosensors, may help to solve current technical barriers facing LC-based biosensors.

## 6. Summary

In addition to the orientation of LC molecules being easily affected by foreign forces, the change in the color and brightness of LCs can be readily observed by polarized light microscopy. Therefore, LC sensing is expected to develop into an inexpensive, sensitive and portable detection method. After more than 20 years of development, LC biosensors have achieved fruitful results in biological analysis (including detection of viruses, bacteria, proteins, amino acids, DNA, etc.) and chemical analysis (including detection of glucose, folic acid, metal ions, etc.), but more work is still needed to extend from experimental research to practical applications.

Above all, the theoretical research on LC sensing, such as the bending elastic constant of LC, the anchoring energy, the relationship between birefringence and the orientation of LCs, and the relationship between the molecular structures of functionalized substances and LCs, needs to be further discussed. All these topics will be helpful for understanding fundamental molecular-level interactions.

Additionally, the stability and reproducibility of LC biosensors are worthy of research attention due to various influence factors, for instance, the thickness of the LC film, the temperature, and the reaction time. Also, the nonstandardization and nonmechanization of the preparation of biosensors cause poor stability and reproducibility.

Moreover, different types of LCs remain to be explored in the development of LC-based sensors besides the most commonly used 5CB.

Furthermore, the various innovative LC-based biosensors have a large space to be explored, for instance, utilizing the electrical, electro-optical, and dielectric property of LC to develop novel sensors.

LC-based biosensors mostly remain in the stage of semi-quantitative measurement at present. The continuous and quantitative analysis of target substances has not been achieved. Therefore, establishment of mathematical model between the color and brightness of LC film with the concentration of target substances to realize their quantitative analysis could be a future issue.
